# A prognostic model using FIGO 2018 staging and MRI-derived tumor volume to predict long-term outcomes in patients with uterine cervical squamous cell carcinoma who received definitive radiotherapy

**DOI:** 10.1186/s12957-023-03116-4

**Published:** 2023-07-21

**Authors:** Lele Zang, Qin Chen, An Lin, Jian Chen, Xiaozhen Zhang, Yi Fang, Min Wang

**Affiliations:** 1 Department of Gynecology, Fujian Medical University Cancer Hospital, FujianCancer Hospital, Fuzhou, China; 2 Department of Radiology, The Affiliated People’s Hospital of Fujian University of Traditional Chinese Medicine, Fuzhou, China

**Keywords:** FIGO 2018, Prognostic model, MRI, Uterine cervical squamous cell carcinoma, Radiotherapy

## Abstract

**Background:**

Uterine cervical carcinoma is a severe health threat worldwide, especially in China. The International Federation of Gynecology and Obstetrics (FIGO) has revised the staging system, emphasizing the strength of magnetic resonance imaging (MRI). We aimed to investigate long-term prognostic factors for FIGO 2018 stage II–IIIC2r uterine cervical squamous cell carcinoma following definitive radiotherapy and establish a prognostic model using MRI-derived tumor volume.

**Methods:**

Patients were restaged according to the FIGO 2018 staging system and randomly grouped into training and validation cohorts (7:3 ratio). Optimal cutoff values of squamous cell carcinoma antigen (SCC-Ag) and tumor volume derived from MRI were generated for the training cohort. A nomogram was constructed based on overall survival (OS) predictors, which were selected using univariate and multivariate analyses. The performance of the nomogram was validated and compared with the FIGO 2018 staging system. Risk stratification cutoff points were generated, and survival curves of low-risk and high-risk groups were compared.

**Results:**

We enrolled 396 patients (training set, 277; validation set, 119). The SCC-Ag and MRI-derived tumor volume cutoff values were 11.5 ng/mL and 28.85 cm^3^, respectively. A nomogram was established based on significant prognostic factors, including SCC-Ag, poor differentiation, tumor volume, chemotherapy, and FIGO 2018 stage. Decision curve analysis indicated that the net benefits of our model were higher. The high-risk group had significantly shorter OS than the low-risk group in both the training (*p* < 0.0001) and validation sets (*p* = 0.00055).

**Conclusions:**

Our nomogram predicted long-term outcomes of patients with FIGO 2018 stage II–IIIC2r uterine cervical squamous cell carcinoma. This tool can assist gynecologic oncologists and patients in treatment planning and prognosis.

## Introduction

Uterine cervical carcinoma ranks fourth in malignant tumors among women worldwide, after breast cancer, colorectal cancer, and lung cancer. In China, it is also the fourth leading cause of cancer death among women and is the most prevalent cancer in women [1]. Most uterine cervical carcinomas are squamous cell carcinomas, and the main therapies are surgery, radiotherapy, and chemotherapy. However, surgery is recommended only for early-stage patients, and most patients are at advanced stages when diagnosed; thus, radiotherapy and chemoradiotherapy play a crucial part in the treatment of these patients [2].

The International Federation of Gynecology and Obstetrics (FIGO), a global professional organization, developed a staging system for widely accepted independent prognostic risk factors used in clinical practice. The FIGO staging system is a clinical staging system based primarily on physical examination findings. In the FIGO 2009 staging system, lymph node metastasis was not included as a prognostic factor, despite being proven by several studies to be a strong prognostic factor for uterine cervical carcinoma [3, 4]. Therefore, the FIGO staging system was revised in 2018 and 2019 to account for lymph node status and emphasize the role of imaging in prognosis [5, 6]. While the FIGO 2018 staging system improved discriminatory power for stages I and IV [7], it did not improve discriminatory power for other stages, especially for stage IIIC [8]. This could be because tumor volume was not included in staging criteria, despite its significant correlation with prognosis even in the evaluation of same-stage tumors [9].

In the FIGO 2018 staging system, magnetic resonance imaging (MRI) plays a central role in uterine cervical cancer staging because it provides excellent contrast resolution, especially for soft tissue, and offers multiparametric imaging. Therefore, it has the advantages of delineating tumor extent and discriminating lymph node metastasis with high accuracy [10–12]. Numerous studies have shown that tumor diameter is an independent prognostic risk factor for uterine cervical carcinoma [13–15]. However, for a malignant tumor, estimation of the tumor volume based only on a single diameter produces inaccurate results because of its complicated and irregular shape. Kim et al. demonstrated that MRI-derived pretreatment tumor volume, but not pretreatment tumor diameter, was significantly correlated with the prognosis of patients with uterine cervical carcinoma who received concurrent chemotherapy and radiotherapy [16].

Squamous cell carcinoma antigen (SCC-Ag), which was discovered by Kato and Torigoe, is a characteristic biomarker for squamous cell carcinoma [17]. The expression level emerges synchronously with the squamous formation of the uterine cervix and increases during the neoplastic transformation of the cervical squamous epithelium [18]. SCC-Ag levels are elevated in 28–88% of patients with uterine cervical squamous cell carcinoma [19]. However, the ability of SCC-Ag to predict the prognosis of uterine cervical carcinoma remains controversial, with some researchers unable to demonstrate any predictive ability of the parameter at all [20]. In contrast, several researchers have demonstrated that SCC-Ag levels alone or in combination with other factors were significantly correlated with the prognosis and even had the capacity to predict the efficacy of treatment or risk of recurrence [21–24]. Thus, we sought to explore the role of SCC-Ag in the present study.

A nomogram can convert complicated results of multivariate analyses into a visual graph that is simple and easy to understand. Many studies have established a prognostic model using a nomogram for patients with uterine cervical carcinoma who received definitive radiotherapy [25, 13, 26]. However, these studies did not use the latest FIGO 2018 staging system.

This study aimed to investigate the long-term prognostic factors for patients with uterine cervical squamous cell carcinoma who received definitive radiotherapy and develop a model to predict prognosis in the context of the FIGO 2018 staging system and the widespread application of MRI.

## Methods

### Participants

**Fig. 1:**
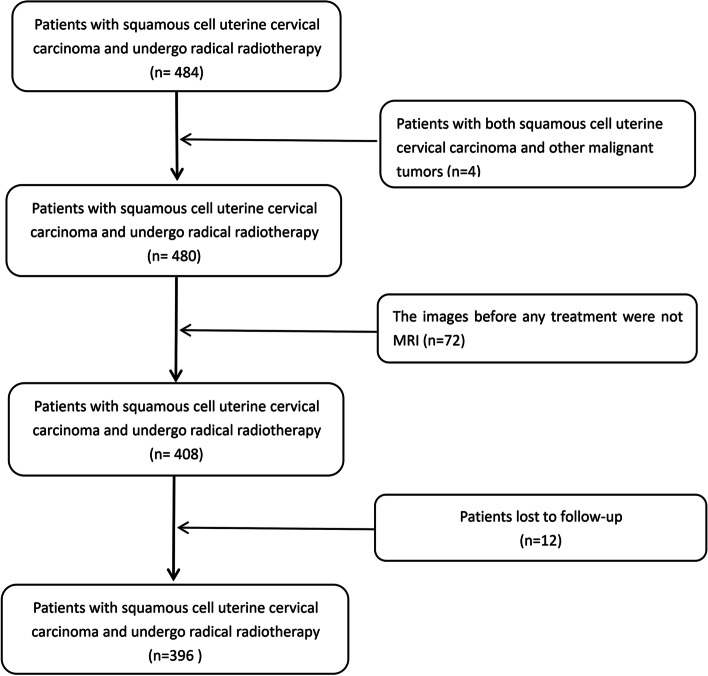
Flowchart for selecting patients

We reviewed the medical records of patients with uterine cervical carcinoma who were diagnosed between 2013 and 2014 and received definitive radiotherapy in our institute. Patients with histologically confirmed squamous cell carcinoma of the uterine cervix that was restaged as II-IIIC2r according to the FIGO 2018 staging system received definitive radiotherapy and underwent an enhanced abdominopelvic MRI scan prior to treatment were enrolled (the flowchart is shown in Fig. 1). Patients with any of the following circumstances were excluded from the study: radiographic evaluation other than MRI, missing data, secondary cancer, or refusal to participate in this study. Overall survival (OS) was defined as the time between diagnosis and death.

### SCC-Ag detection

Fasting venous blood was taken from patients in the morning before treatment, and serum was separated by centrifugation within 4 h. Abbott Diagnostics I2000 automatic chemiluminescence immunoassay analyzer and supporting reagents were used to detect SCC-Ag. The operation was carried out in strict accordance with the instrument’s operation specifications and the kit’s instructions. Since serum SCC-Ag level was not increased in all patients with cervical squamous cell carcinoma [19], was also elevated in other types of cancers [27, 28], and was affected by reduced kidney function [29], the sensitivity and specificity were both unsatisfactory. Thus, we aimed to explore the prognostic value of SCC-Ag combined with other clinical factors.

### MRI analysis

A superconducting MRI scanner (Signa 1.5 T Excite iii HD, GE) was used to obtain the images before any treatment was started. The MRI scan sequences included fast spin echo (FSE), T1-weighted imaging (T1WI), FSE T2-weighted imaging (T2WI), diffusion-weighted imaging (DWI), and coronal FSE T2WI. DWI was performed using a spin echo plane echo sequence. After plain scanning, gadopentetic acid was injected at 3 mL/s through the cubital vein at 0.1 mmol/kg, and then axial, sagittal, and coronal scans using a liver acquisition with volume acceleration sequences were carried out on gradient echo T1WI of 3D volumetric interpolation. On axial T2WI images showing the largest tumor, tumor length was defined as the largest diameter in the left–right direction and tumor width as the vertical diameter in the anterior–posterior direction. On sagittal T2WI images showing the largest tumor, tumor thickness was defined as the longest diameter in the foot–head direction. The tumor volume was calculated using the following equation: *π* × tumor length (cm) × tumor width (cm) × tumor thickness (cm)/6 = V. Enlarged lymph nodes with short diameters of > 1 cm were considered to be metastatic lymph nodes. All MRI data and medical records were retrospectively reviewed and confirmed by two radiologists with more than 10 years of experience in interpreting gynecologic oncology images.

### Definitive radiotherapy

All patients received definitive radiotherapy. Radiotherapy volume, dose, and protocol were confirmed with a central review by gynecological oncologists Qin Chen and Min Wang. Radiotherapy in this study consisted of two parts: external beam radiotherapy (EBRT) and brachytherapy (BT). In EBRT, some patients received conventional radiotherapy, while others received intensity-modulated radiotherapy, which was administered through 6 MV photons once a day, 5 days a week. The prescription dose of EBRT was 45–56 Gy at 1.8–2.0 Gy per fraction. Patients with FIGO 2018 stage IIIc2r or IIIc1r with common iliac lymph node metastasis received extended-field irradiation, which included the whole pelvic and para-aortic lymph node area. Since BT was superior to inversely planned EBRT in both target doses and organs at risk (OAR) sparing, BT is irreplaceable in radical radiotherapy of locally advanced cervical carcinoma [30]. All these patients received high-dose-rate BT, which was started 2 weeks after the beginning of EBRT and was performed once a week, with the A point dose of 7 Gy per fraction. After the initial EBRT and BT was completed, evaluations were separately conducted by two gynecological oncologists. Patients were treated with an additional 1 or 2 times of BT, depending on the time of radiotherapy being more than 8 weeks, tumor regression, and tolerance doses of OAR. BT was administered 3–7 times. The overall treatment time of radical radiotherapy was controlled within 56 days, as much as possible. Subsequently, both the EBRT and BT doses were converted into 2-Gy equivalent doses (EQD_2_), respectively, using the following linear-quadratic model: prescription dose × (*α*/*β* + fractionated dose) / (*α*/*β* + 2) = EQD_2_, where *α*/*β* = 10. The EQD_2_ for EBRT and BT were then summed up to obtain the dose for analysis in this study.

### Chemotherapy

Most patients in this study received chemotherapy, except for those who were older, had contraindications, and refused to receive it. These patients also received different chemotherapy regimens. Fourteen patients received platinum monotherapy with concurrent chemoradiation therapy. A total of 300 patients received platinum-based dual drug chemotherapy, of which 7 patients used docetaxel due to paclitaxel allergy, and the remaining 293 patients used paclitaxel. Two patients received intravenous gemcitabine and platinum chemotherapy due to taxane allergy. To stop massive vaginal bleeding, 8 patients with bulky tumors received interventional chemotherapy with paclitaxel, cisplatin, and bleomycin. Considering these scenarios, we included the presence of chemotherapy as a factor in the analysis.

### Statistical analysis

Patient information was anonymized prior to analysis. Using computer-generated random numbers, we grouped the patients into training and validation cohorts at a ratio of 7:3. The function “surv_cutpoint” in the R package “survminer” was implemented to generate the optimal cutoff values of SCC-Ag and tumor volume for the training cohort [31]. Continuous parameters presented as means ± standard deviation or medians with interquartile ranges were compared between the training and validation sets using Student’s *t*-test or the Mann–Whitney *U* test, as appropriate. The chi-squared test or Fisher’s exact test was used to compare the frequency distribution of categorized parameters.

A nomogram was constructed based on significant predictors of overall survival (OS) selected by multivariate Cox proportional hazards regression using a stepwise selection method that included variables with a *p*-value of < 0.05 in the univariate analysis and of clinical importance. In the nomogram, points were assigned by drawing a line upward from the corresponding values to the “Points” line. The sum of the points, plotted on the “Total Points” line, corresponds to predictions of 3-year, 5-year, and 7-year OS rates in patients with uterine cervical squamous cell carcinoma. Based on the concordance index (C-index) and the area under the time-dependent receiver operating characteristic curve (AUC), the predictive accuracy of the constructed nomogram was evaluated. Calibrating this constructed nomogram, we used bootstraps of 1000 resamples with calibration curves. The performance of the nomogram was internally validated using the validation set and compared with that of the FIGO 2018 staging system. To measure the improvement in the predictive effect of our model, we used the net reclassification improvement (NRI) and integrated discrimination improvement (IDI). It is only possible to evaluate a diagnostic method by assessing its receiver operating characteristic curve from the specificity and sensitivity plot; however, although this is considered an accurate method, the patients are not guaranteed to benefit from it. A method of evaluation devised by Vickers and Elkin, called decision curve analysis (DCA), is capable of calculating the net benefits of the model [32]. In this study, the sum score of each patient was calculated from the nomogram. Subsequently, cutoff points for risk stratification (low and high) were generated using the “surv_cutpoint” function of “survminer.” The log-rank test was used to compare the Kaplan–Meier survival curves of the low-risk and high-risk groups using the training and validation sets. Our statistical analyses were performed using R software (version 4.0.3). We considered a *p*-value of 0.05 to be statistically significant.

## Results

### Patient OS and grouping

A total of 484 patients with uterine cervical squamous cell carcinoma were restaged according to the FIGO 2018 staging system. Four patients who had other malignancies, 72 patients who underwent non-MRI scanning prior to any treatment, and 12 patients who were lost to follow-up were excluded. Finally, 396 patients were enrolled in this study. The date of the last follow-up was December 12, 2021. The median follow-up time was 89.77 months. The 3-year, 5-year, and 7-year OS rates were 87.1%, 83.3%, and 81.8%, respectively.

### Cutoff values, patient characteristics, and results of univariate and multivariate analyses of factors for OS

**Fig. 2:**
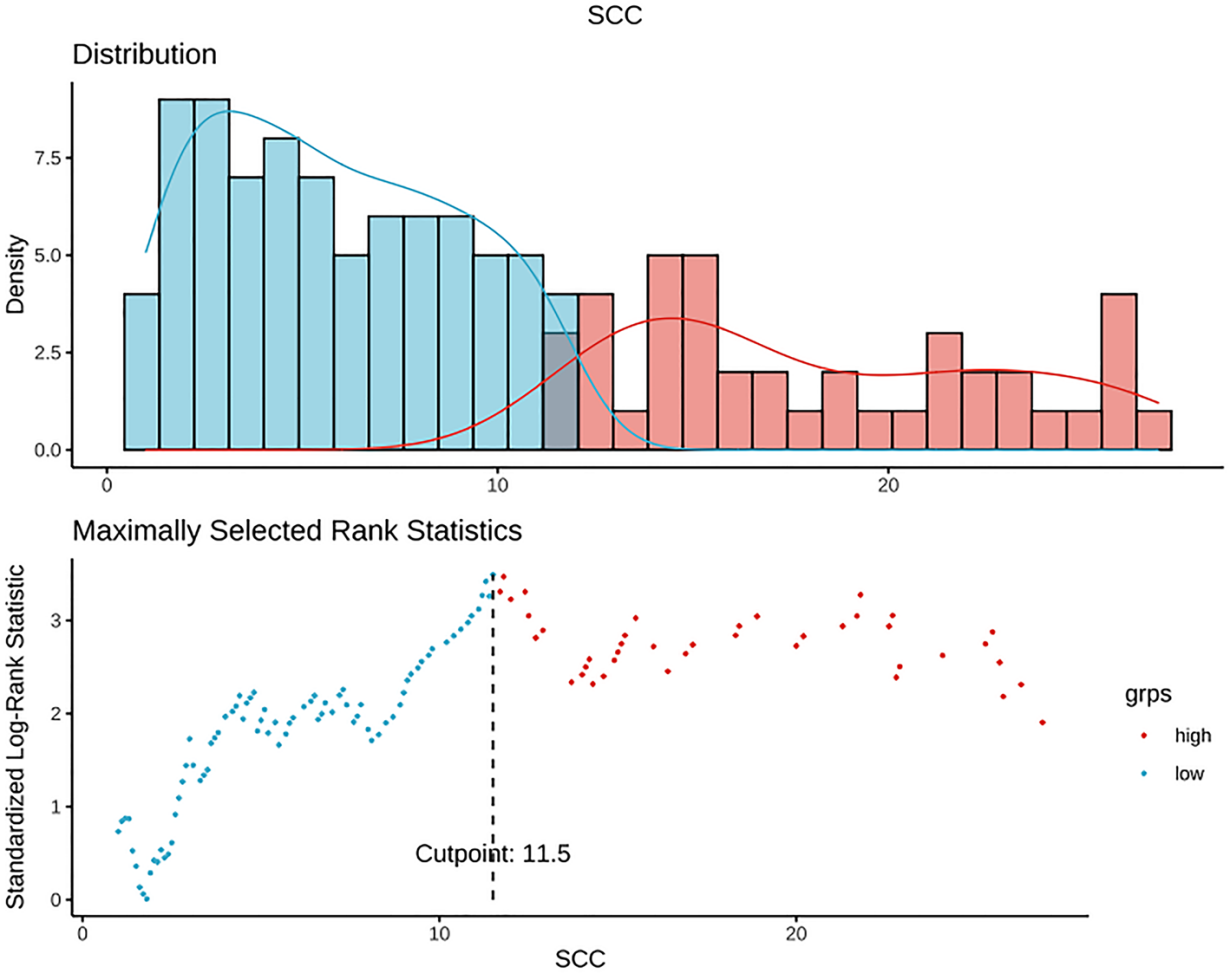
Cut-off point for squamous cell carcinoma antigen (SCC-Ag)

**Fig. 3:**
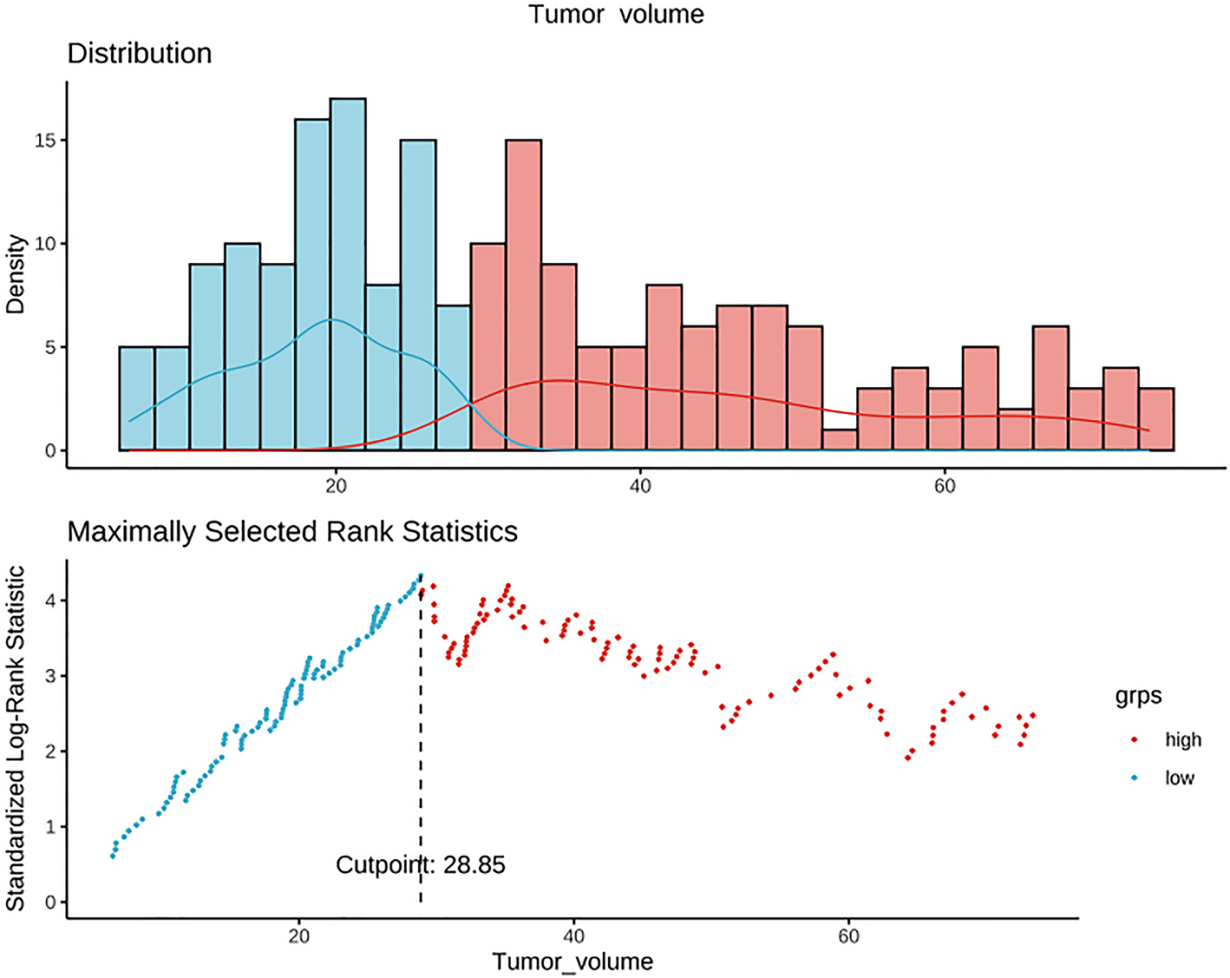
Cut-off value of tumor volume derived from magnetic resonance imaging (MRI)

In total, 277 and 119 patients were randomized to the training and validation sets, respectively. The cutoff value of SCC-Ag derived from the training set was 11.5 ng/mL (Fig. 2). The cutoff value of tumor volume derived from MRI was 28.85 cm^3^ using the same method as that for SCC-Ag (Fig. 3). SCC-Ag and tumor volumes were converted into categorical variables depending on their cutoff values. Patient characteristics in both the training and validation sets are shown in Table 1. There were no significant differences between the training and validation sets in terms of age at diagnosis, SCC-Ag level, MRI-derived tumor volume, radiotherapy modality, presence of chemotherapy, time of radiotherapy, EQD_2_ of point A, histologically poor differentiation, hemoglobin level before treatment, parametrial invasion status, FIGO 2018 stage, and survival status. In the multivariate analysis, SCC-Ag level, histologically poor differentiation, MRI-derived tumor volume, presence of chemotherapy, and FIGO 2018 stage were found to be significant prognostic factors for uterine cervical squamous cell carcinoma (Table 2).

**Table 1:** Patients’ characteristics for both training set and validation set

Variables	Training set *n* = 277	Validation set *n* = 119	*P* value
Age (year, mean ± standard deviation)	55.0 ± 9.5	54.2 ± 9.9	0.4622
SCC-Ag			0.708
≤ 11.5 ng/ml	200 (72.20%)	83 (69.75%)	
11.5 ng/ml	77 (27.80%)	36 (30.25%)	
Tumor volume (cm^3^)			0.6936
≤ 28.85	131 (47.29%)	53 (44.54%)	
> 28.85	146 (52.71%)	66 (55.46%)	
Poor differentiation			0.853
No	229 (82.67%)	100 (84.03%)	
Yes	48 (17.33%)	19 (15.97%)	
Hemoglobin concentration (g/L)			0.4388
≥ 120	209 (75.45%)	83 (69.75%)	
90 g/L ~ 119	46 (16.61%)	26 (21.85%)	
< 90	22 (7.94%)	10 (8.40%)	
Parametrial invasion			0.6113
No	24 (8.66%)	10 (8.40%)	
Parametrial invasion	139 (50.18%)	66 (55.46%)	
Pelvic wall extension	114 (41.16%)	43 (36.13%)	
FIGO2018 stage			0.4027
II	97 (35.02%)	49 (41.18%)	
IIIa and IIIb	64 (23.10%)	19 (15.97%)	
IIIc1r	99 (35.74%)	43 (36.13%)	
IIIc2r	17 (6.14%)	8 (6.72%)	
Chemotherapy			0.5438
No	53 (19.13%)	19 (15.97%)	
Yes	224 (80.87%)	100 (84.03%)	
Radiotherapy modality			0.6598
Conventional radiotherapy	157 (56.68%)	71 (59.66%)	
IMRT	120 (43.32%)	48 (40.34%)	
Time of radiotherapy(day)			0.3521
≤ 56	101 (36.46%)	50 (42.02%)	
> 56	176 (63.54%)	69 (57.98%)	
EQD_2_ dose of point A (Gy)			0.8446
≤ 85	45 (16.25%)	21 (17.65%)	
> 85	232 (83.75%)	98 (82.35%)	
Status			0.6846
Alive/Censored	224 (80.87%)	99 (83.19%)	
Dead	53 (19.13%)	20 (16.81%)	

*SCC-Ag* squamous cell carcinoma antigen, *FIGO* International Federation of Gynecology and Obstetrics, *IMRT* intensity modulated radiation therapy, *EQD*_*2*_ 2-Gy equivalent dose

**Table 2:** Univariate analysis and multivariate Cox proportional hazards regression of training set using a stepwise-selection method

Variables	Univariate analysis		Multivariate analysis	
	Coefficient (95%CI)	*P* value	Coefficient *t*(95%CI)	*P* value
Age	0.99 (0.96–1.02)	0.677		
SCC-Ag (ng/ml)				
≤ 11.5	Reference		Reference	
> 11.5	2.59 (1.51–4.44)	0.001	1.91 (1.07–3.42)	0.028
Tumor volume (cm^3^)				
≤ 28.8	Reference		Reference	
> 28.8	3.89 (2.00–7.56)	0.000	2.84 (1.40–5.74)	0.004
Poor differentiation				
No	Reference		Reference	
Yes	2.33 (1.29–4.18)	0.005	2.81 (1.52–5.19)	< 0.001
Hemoglobin concentration (g/L)				
≥ 120	Reference			
90 g/L ~ 119	2.10 (1.12–3.94)	0.021		
< 90	2.49 (1.10–5.64)	0.029		
Parametrial invasion				
No	Reference			
Parametrial invasion	1.20 (0.36–4.03)	0.764		
Pelvic wall extension	2.19 (0.67–7.18)	0.197		
FIGO2018 stage				
II	Reference		Reference	
IIIa and IIIb	1.99 (0.79–5.05)	0.146	1.42 (0.55–3.70)	0.469
IIIc1r	3.80 (1.73–8.34)	0.001	2.45 (1.07–5.60)	0.034
IIIc2r	6.20 (2.25–17.12)	0.000	4.26 (1.49–12.17)	0.007
Chemotherapy				
No	Reference		Reference	
Yes	0.71 (0.38–1.32)	0.277	0.50 (0.27–0.96)	0.037
Radiotherapy modality				
Conventional radiotherapy	Reference			
IMRT	1.26 (0.74–2.16)	0.399		
Time of radiotherapy(day)				
≤ 56	Reference			
> 56	1.37 (0.76–2.47)	0.289		
EQD_2_ dose of point A (Gy)				
≤ 85	Reference			
> 85	1.60 (0.69–3.75)	0.277		

*SCC-Ag* squamous cell carcinoma antigen, *FIGO* International Federation of Gynecology and Obstetrics, *IMRT* intensity modulated radiation therapy, *EQD*_*2*_ 2-Gy equivalent dose

### Establishment and evaluation of the prognostic model

**Fig. 4:**
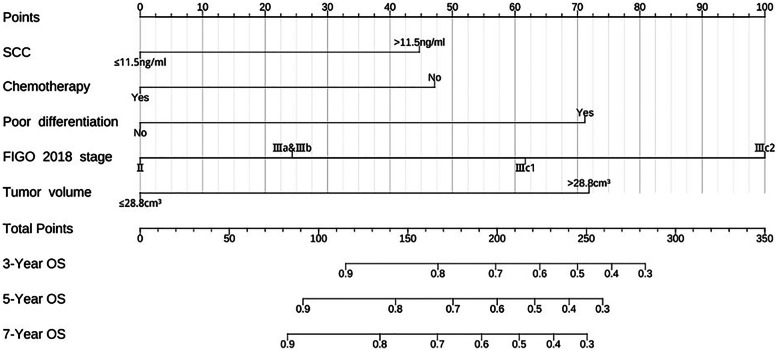
Nomogram to predict 3-year, 5-year, and 7-year overall survival (OS) of uterine cervical squamous cell carcinoma received definitive radiotherapy

**Fig. 5:**
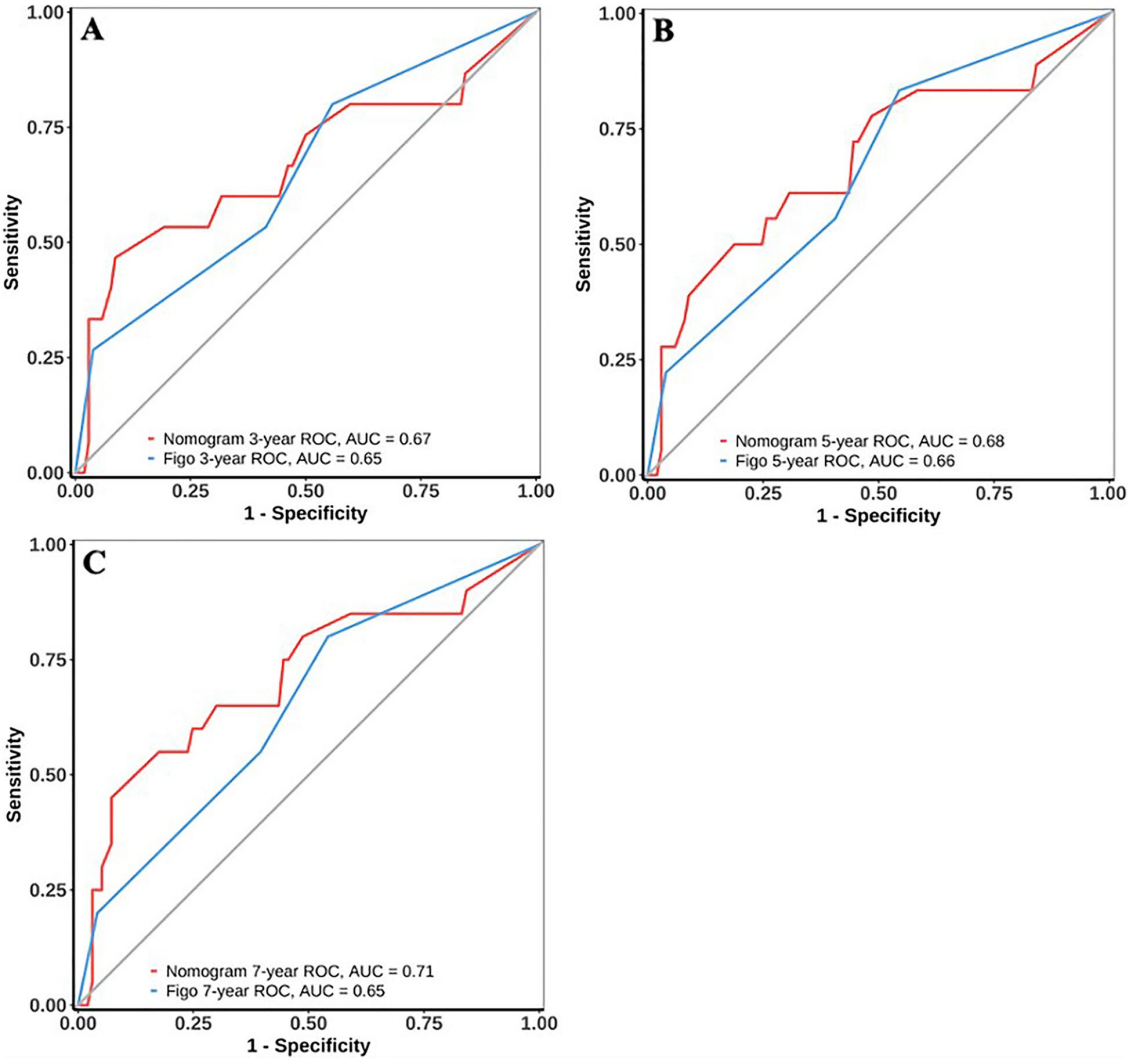
The area under the time-dependent receiver operating characteristic curves for 3-year, 5-year and 7-year OS from the validation set. **A** AUC for 3-year OS. **B** AUC for 5-year OS. **C** AUC for 7-year OS. OS overall survival, AUC area under the curve, FIGO International Federation of Gynecology and Obstetrics

**Fig. 6:**
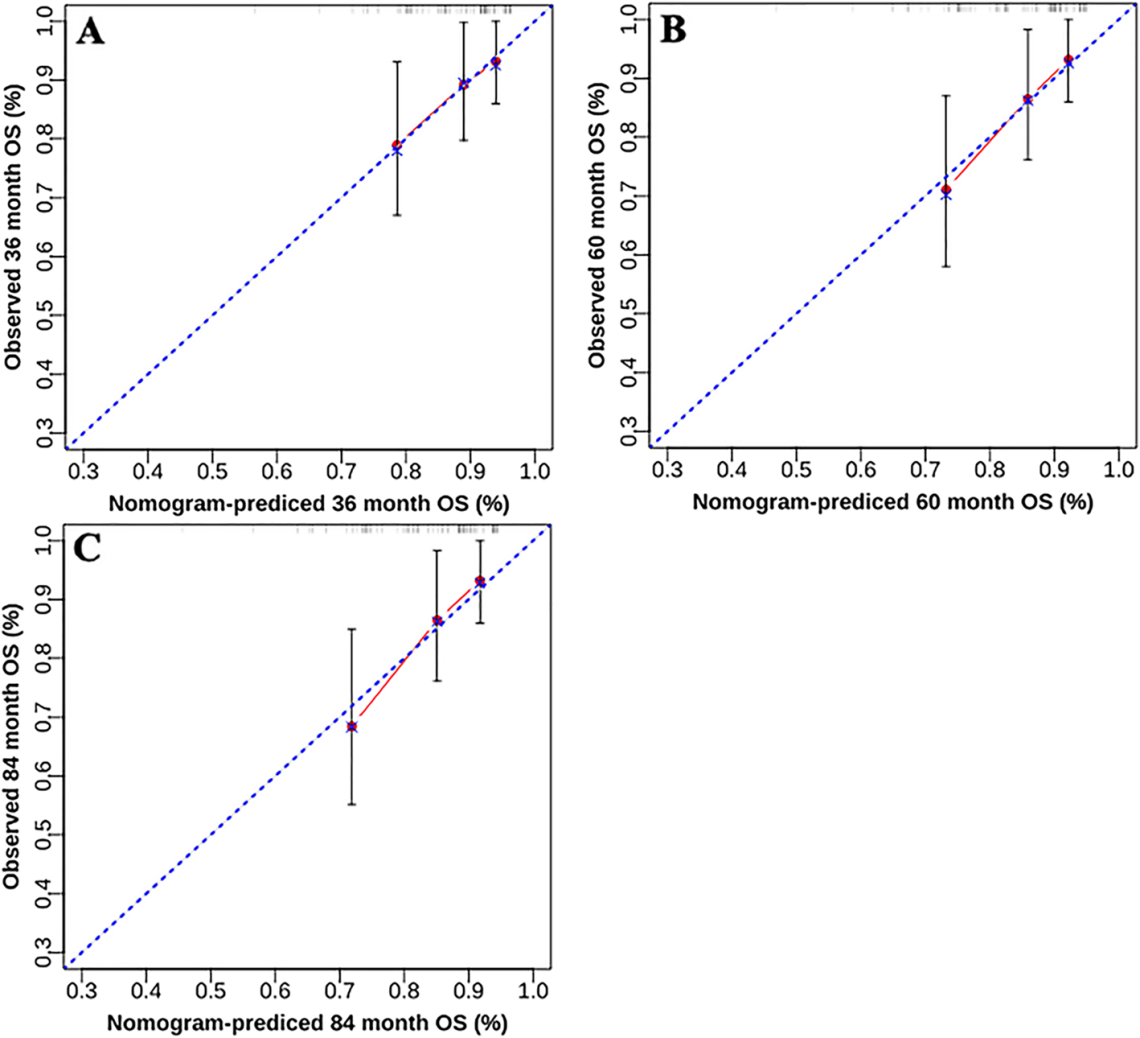
Calibration plots for 3-year, 5-year, and 7-year OS from the validation set. **A** Calibration plots for 3-year OS. **B** Calibration plots for 5-year OS. **C** Calibration plots for 7-year OS. OS overall survival, FIGO International Federation of Gynecology and Obstetrics

**Fig. 7:**
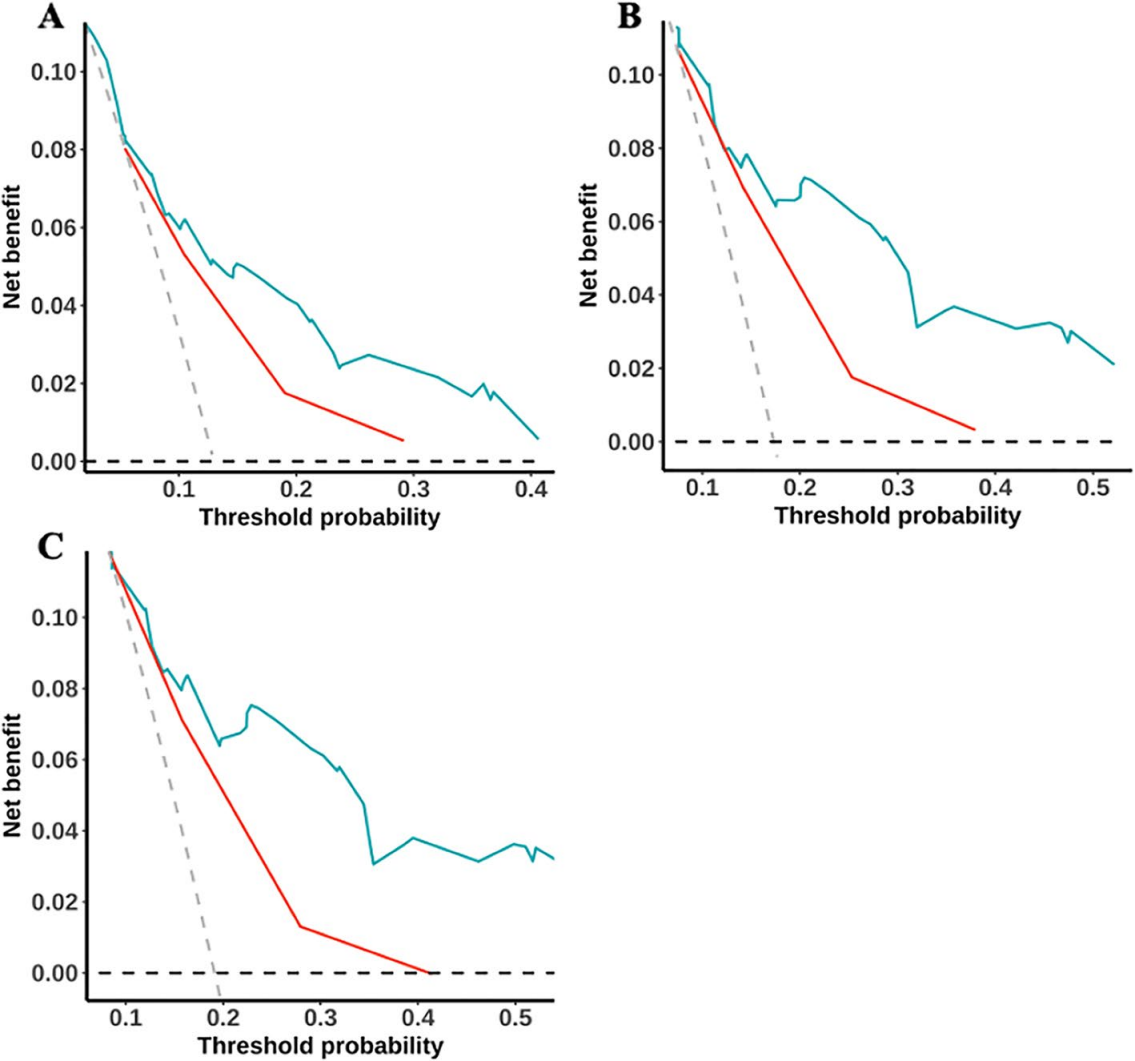
Decision curve analysis (DCA) for our nomogram and FIGO 2018 staging system. **A** DCA for 3-year OS. **B** DCA for 5-year OS. **C** DCA for 7-year OS.DCA decision curve analysis. OS overall survival, FIGO International Federation of Gynecology and Obstetrics

The established nomogram for predicting OS is shown in Fig. 4. The corresponding score can be calculated through the top “Points” line of the nomogram for each prognostic factor, and then the 3-year, 5-year, and 7-year OS could be estimated by summing up the individual scores and checking the “Total Points” line at the bottom. The C-indices of our nomogram for the training and validation sets were 0.74 (95% confidence interval [CI]: 0.67–0.80) and 0.70 (95% CI: 0.57–0.82), while those of the FIGO 2018 staging system were 0.66 (95% CI: 0.59–0.73) and 0.63 (95% CI: 0.52–0.75), respectively (Table 3). For our model, the AUCs of the validation set for 3-year, 5-year, and 7-year OS were 0.67, 0.68, and 0.71, respectively. Meanwhile, the corresponding AUCs of the FIGO 2018 staging system were 0.65, 0.66, and 0.65. Notably, our model had higher C-indices and AUCs than the FIGO 2018 staging system, suggesting that our model has a better discrimination ability than the FIGO staging system (Fig. 5). Calibration curves with bootstraps of 1000 resamples also indicated a good agreement between the predicted OS and observed outcomes (Fig. 6). As shown in Table 4, the NRI values of our nomogram for 3-year, 5-year, and 7-year OS were 0.21, 0.38, and 0.38, respectively, in the validation set (all *p* < 0.01); the corresponding IDI values were 0.08, 0.14, and 0.10 (all *p* < 0.01). These values suggest that the predictive performance of our nomogram was substantially improved. DCA indicated that the net benefits of our model were higher than those of the FIGO staging system at 3, 5, and 7 years (Fig. 7).

**Table 3:** Comparison of C-index for our model and FIGO2018 stage system

Group	Nomogram (95%CI)	FIGO2018 (95%CI)
Training set	0.74 (0.67–0.80)	0.66 (0.59–0.73)
Validation set	0.70 (0.57–0.82)	0.63 (0.52–0.75)

*FIGO* International Federation of Gynecology and Obstetrics

**Table 4:** The NRI and IDI of the nomogram in overall survival prediction for squamous cell uterine cervical carcinoma patients received definitive radiotherapy compared with FIGO2018 stage system in validation set

Index	Estimate	95% CI	*P* value
NRI (vs. FIGO2018 stage)			
For 3-year OS	0.21	0.07–0.36	< 0.01
For 5-year OS	0.38	0.23–0.53	< 0.001
For 7-year OS	0.38	0.20–0.56	< 0.001
IDI (vs. FIGO2018 stage)			
For 3-year OS	0.08	0.03–0.13	< 0.01
For 5-year OS	0.14	0.08–0.19	< 0.001
For 7-year OS	0.10	0.005–0.15	< 0.001

*FIGO* International Federation of Gynecology and Obstetrics, *NRI* Net reclassification improvement, *IDI* Integrated discrimination improvement, *OS* Overall survival

### Risk stratification by the nomogram

**Fig. 8:**
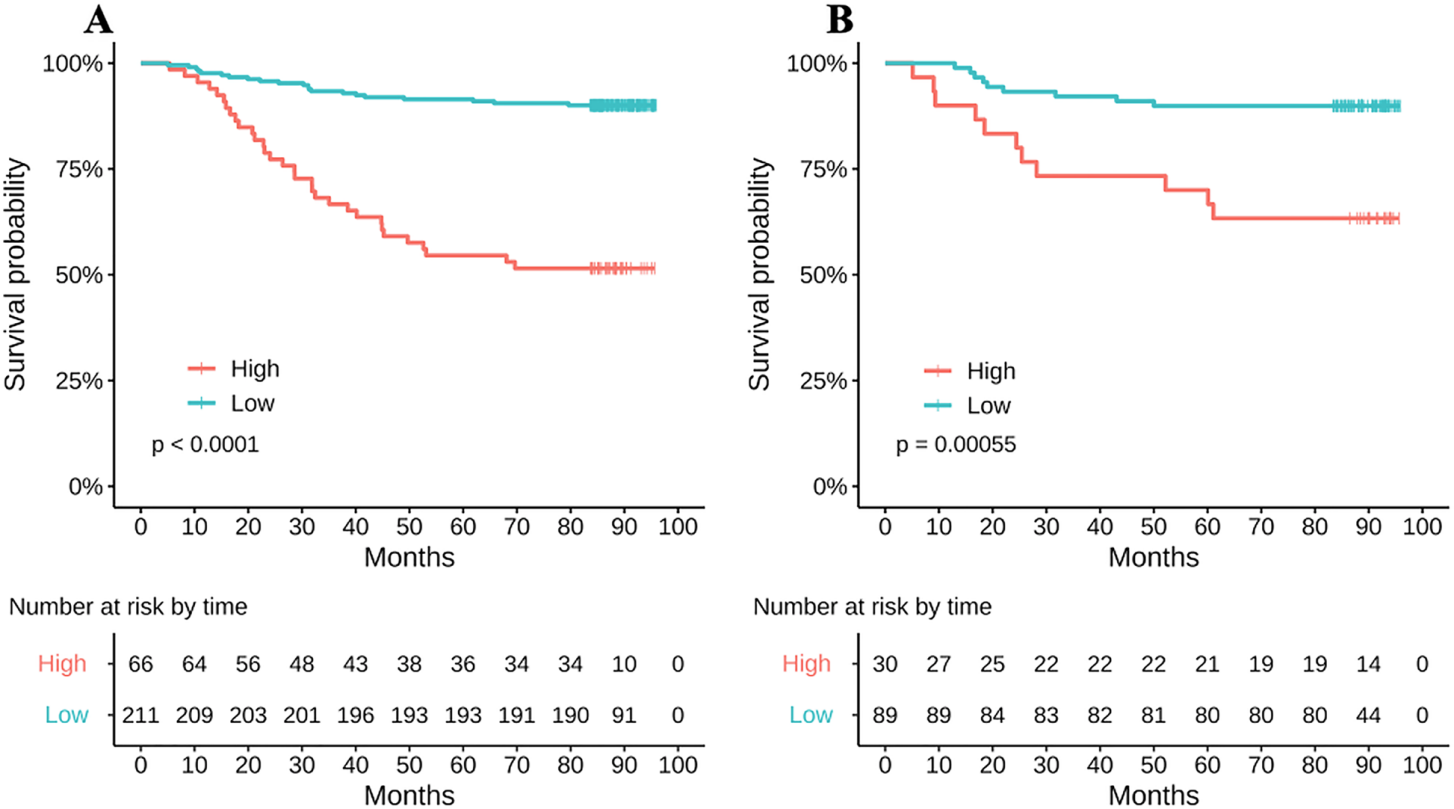
Survival curves of low-risk and high-risk groups using the training and validation sets. **A** Curve of survival of the training cohort. **B** Curve of survival of the validation cohort

The cutoff point for risk stratification (low and high) was 171.21, based on the scores of patients in the training group. The OS of patients in the high-risk group was significantly shorter than that in the low-risk group in both the training (*p* < 0.0001) and validation sets (*p* = 0.00055) (Fig. 8).

## Discussion

We established a prognostic model to predict the long-term OS of patients with FIGO 2018 stage II-IIIC2r uterine cervical squamous cell carcinoma who received definitive radiotherapy. In this study, we found that factors such as the SCC-Ag level, histologically poor differentiation, tumor volume derived from MRI prior to treatment, FIGO 2018 stage, and presence of chemotherapy were predictors of prognosis in these patients.

We found that uterine cervical squamous cell carcinoma with elevated pretreatment serum SCC-Ag concentrations of more than 11.5 ng/ml had a significantly worse prognosis than those with concentrations less than or equal to 11.5 ng/ml. The outcomes from some other studies were in line with ours. Cheng et al. found that pretreatment SCC Ag >10 ng/mL was a significant poor prognostic factor of progression-free survival (PFS), locoregional recurrence-free survival, and distant metastasis-free survival in patients with stage IB-IVA cervical cancer in patients who underwent concurrent chemoradiation therapy [33]. Chen et al. enrolled 203 patients with stage IIA–IVA cervical squamous cell carcinoma and found in their study that pretreatment SCC >11.4 ng/mL was an independent predictor of PFS [34]. Apparently, increased SCC-Ag level was associated with a worse patient outcome [35]. The underlying reason for this may be that SCC-Ag could promote radioresistance of tumor cells by suppressing radiation-induced cell death [36]. Also, Shou et al. found that high SCC-Ag (≥14.6 ng/mL) was associated with the FIGO 2018 stage [37], with a more advanced stage indicating a poorer prognosis.

Brambs et al. reexamined the histological slides of 467 patients with surgically treated FIGO stage IB1–IIB uterine cervical carcinoma and found that binary grading (grade 1/2 vs. grade 3) may be more suitable for evaluating prognostic survival than conventional tumor grading based on the degree of keratinization [38]. This is also the reason we only considered poor differentiation, and not tumor grade, in our analysis, and our results are consistent with those of other studies. Studies by Xie et al. and Luo et al. showed that patients with poor differentiation (grade 3) had a significantly worse OS than those with grade 1/2 uterine cervical carcinoma in the early stage (FIGO stages IA2–IIB) [39, 40]. Using data from 31,536 women with uterine cervical squamous cell carcinoma extracted from the Surveillance, Epidemiology, and End Results (SEER) Program between 1983 and 2013, Matsuo et al. found that grade 3 tumors (poor differentiation) were independently associated with decreased cause-specific survival, especially among patients with stage II–III disease [41]. These findings could be attributed to the keratin pattern being a component of aggregated cervical squamous cell carcinoma related to survival [42]. On the contrary, Kumar et al. analyzed patients who were diagnosed with uterine cervical squamous cell carcinoma between 1988 and 2004 using limited data from the SEER Program and figured out that nonkeratinized squamous cell carcinoma, rather than keratinized squamous cell carcinoma, might be more radiosensitive and associated with a better prognosis [43]. Notably, the racial composition of the Asian population was 11.4%, and the proportion of poorly differentiated cases in our study was approximately 16%—a reason why the results of the above-mentioned studies are different from ours and why our findings are only applicable to Chinese patients.

FIGO 2018 is a clinical staging system based on physical examination and imaging. Gynecologists rely heavily on physical examination when evaluating primary tumors. However, palpation as a component of physical examination is a subjective method that can only determine the axial diameter of the tumor but cannot estimate the contribution of normal cervical tissue. Thus, clinical estimation of tumor size through palpation cannot adequately represent the actual tumor volume [44]. Narayan et al. demonstrated in their study that tumor volume measured using MRI accurately reflected the extent of local disease and could be used as an objective measurement of the primary site of cervical cancer [45]. Other researchers also demonstrated that an increase in tumor volume is associated with lymph node metastasis and poor prognosis [46, 47]. Some investigators have even observed that MRI-derived tumor volume provides more accurate and useful prognostic information than that provided by the FIGO staging system [48]. However, not all these studies used the FIGO 2018 staging system. In our results, MRI-derived tumor volume was a critical prognostic factor for FIGO 2018 stage II–IIIC2r uterine cervical squamous cell carcinoma.

The FIGO staging system is widely used in the clinical management of uterine cervical carcinoma and is a paramount factor affecting the treatment outcome. However, there are other prognostic factors that must be considered. Lymph node metastasis could strongly decrease the survival of patients with uterine cervical carcinoma, and in this regard, the FIGO 2018 staging system defined stage IIIC1 as pelvic lymph node metastasis and stage IIIC2 as para-aortic lymph node metastasis, both of which can be suffixed with the letter “r” or “p” to refer to a radiological or pathological finding, respectively [5]. Therefore, we contrasted a nomogram using the FIGO 2018 staging system and other clinical factors, with emphasis on MRI-derived tumor volume. NRI and IDI are indices indicating how a model’s predictive power improves after a new risk factor(s) is introduced. A value of > 0 indicates improvement. In this study, the NRI and IDI values for 3-year, 5-year, and 7-year OS were all > 0, suggesting that our model achieved a better predictive ability than the FIGO 2018 staging system. Thus, our nomogram could offer patients accurate individual predictions.

Moreover, synchronous radiotherapy and platinum-based chemotherapy is the standard treatment for locally advanced cervical cancer. Indeed, chemotherapy is thought to act as a radiosensitizer with the aim of eradicating occult metastases [49]. Notably, most patients enrolled in our study received platinum- and taxane-based dual drug chemotherapy. Our results confirmed that chemotherapy may improve the OS of patients, and chemotherapy was included in our model as a therapeutic factor. Garces et al. demonstrated that paclitaxel–carboplatin is an active and well-tolerated regimen for the treatment of advanced cervical cancer [50]. Moreover, a multicenter phase II trial conducted by Takekuma et al. demonstrated that intravenous paclitaxel and nedaplatin in patients with advanced/recurrent uterine cervical cancer exhibited favorable antitumor activity [51]. In addition, a systematic review and meta-analysis involving 17 published studies and 4106 patients identified that concurrent chemoradiotherapy with platinum-based dual drug therapy improved OS and PFS of locally advanced cervical carcinoma patients relative to concurrent chemoradiotherapy with platinum monotherapy [52]. All of above findings are consistent with our observations. This chemotherapy parameter could be very useful both to gynecologic oncologists when creating treatment plans and to patients during decision-making for accepting those plans. For instance, if a virtual 59-year-old patient with a histologically confirmed uterine cervical squamous cell carcinoma with FIGO stage IIIb, pretreatment SCC-Ag level of 15.0 ng/mL, and MRI-derived tumor volume of 35 cm^3^ decides to undergo chemotherapy, the total score for all parameters calculated from the nomogram will be 139, and the predictive 3-year, 5-year, and 7-year OS will be 86%, 80%, and 78%, respectively. However, if the patient refuses to receive chemotherapy, the total score will be 187, and the predictive 3-year, 5-year, and 7-year OS will be 73%, 63%, and 60%. In this example, radiotherapy without chemotherapy is associated with an apparent decrease in OS.

There are several nomograms established by other researchers for predicting uterine cervical carcinoma prognosis following radiotherapy [26, 53, 54]. Other researchers explored the predictive accuracy of the FIGO 2018 staging system and other significant prognostic factors; however, they have not investigated the value of MRI-derived tumor volume in predicting prognosis of these patients [55, 56]. To our knowledge, this nomogram is the first long-term model for predicting OS in patients with uterine cervical squamous cell carcinoma who received definitive radiotherapy using the FIGO 2018 staging system and pretreatment tumor volume derived from MRI.

This study has some limitations that should be acknowledged. First, it was a retrospective study and is therefore prone to selection bias. However, several measures were taken to minimize selection bias. From study design to implementation, we selected patients with cervical squamous cell carcinoma that were treated in our hospital from 2013 to 2014. Moreover, all case data were collected from electronic data. During this period, there were no significant changes in the treatment methods for cervical cancer, and long-term follow-up was conducted on the patients. Finally, during the analysis process, the patients were randomly divided into the training and a validation sets, with balanced baseline data. Second, the chemotherapy regimens in this study were heterogeneous.

Specific chemotherapy schemes included platinum single drug chemotherapy, platinum and taxane dual drug chemotherapy, such as paclitaxel and platinum, docetaxel and platinum, gemcitabine and platinum dual drug chemotherapy, as well as paclitaxel, cisplatin, and bleomycin interventional chemotherapy scheme. As a result, we only analyzed the presence of chemotherapy as a factor in our research and found that chemoradiotherapy could improve the OS of patients compared to radiotherapy alone. Nevertheless, further stratified analysis of the specific chemotherapy regimens is necessary. Third, radiotherapy plays a significant role in the treatment of cervical cancer. Compared with traditional 2D radiation therapy, intensity modulated radiotherapy (IMRT) has dosimetric advantages in organ preservation and has made possible safer dose escalation especially to the para-aortic region. This provides better clinical outcomes while reducing toxicity [57–59]. However, we did not observe a significant impact of IMRT on survival in our study. The underlying reason for this may be that patients with lymph node metastasis were more likely to receive IMRT, while patients without lymph node metastasis were more likely to receive conventional radiation therapy. FIGO 2018 stage of patients with lymph node metastasis were more advanced, this may have led to the effectiveness of IMRT being underestimated. Fourth, although our prediction model was developed using data from a single institution, the presented results are based on a follow-up period of 89.77 months. In addition, all the predictive factors included in this study are easy to obtain. Therefore, external validation for our findings at multiple centers is clinically feasible. However, patients with cervical squamous cell carcinoma have a relatively good prognosis, and hence our research focused on the long-term survival; this requires several years of follow-up. Therefore, we plan to perform this analysis in a future study.

## Conclusions

We established a nomogram using MRI-derived tumor volume to predict the long-term outcomes of patients with FIGO 2018 stage II–IIIC2r uterine cervical squamous cell carcinoma. The tool may be useful to gynecologic oncologists when creating treatment plans and predicting individual prognoses and to patients when making treatment decisions.

## Data Availability

The datasets used and analyzed during the current study are available from the corresponding author on reasonable request.
